# Supplementary material for: Genome-wide modelling of plant transcription factor binding captures regulatory variants associated with phenotypic traits
Source: Nat Commun. 2026 Jun 3;17:4913. doi: 10.1038/s41467-026-73634-8 (PMC13234004; doi:10.1038/s41467-026-73634-8)
Supplement: Supplementary file 1 — Supplementary Information [file 41467_2026_73634_MOESM1_ESM.pdf]

Genome-wide modelling of plant transcription factor binding captures regulatory variants associated with phenotypic traits

Peleke *et al.*

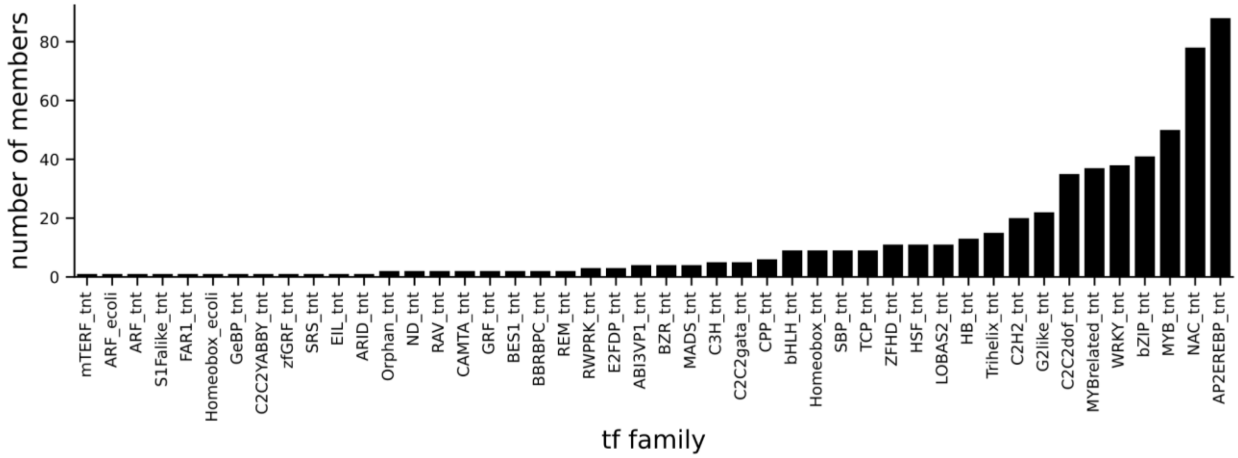

Supplementary Figure 1. Number of members per transcription factor family with recorded DAP-seq peaks used for the training of the multi-label classification model. There are in total 219 ampDAP-seq and 349 DAP-seq datasets in the narrowPeak format with a fraction of reads in peaks (FRiP) value ≥ 5% (http://neomorph.salk.edu/dap_web/pages/browse_table_aj.php) [1].

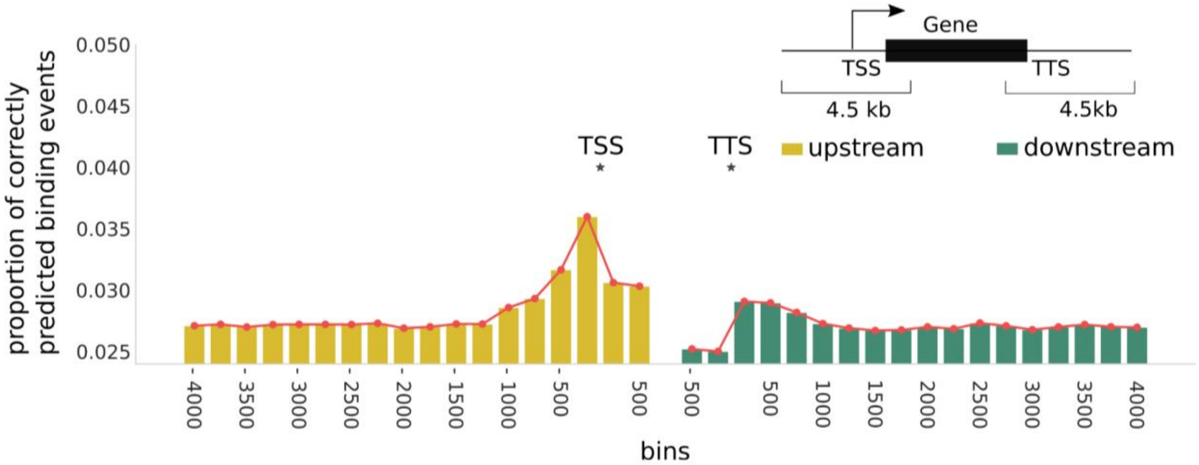

Supplementary Figure 2. Distribution of correctly predicted binding events within 4 kbp intergenic regions up- and downstream of the transcription start and termination site (TSS, TTS), respectively, along with 0.5 kbp internal regions up and downstream of genes. The sequence regions are binned into 250 bp sub-windows to meet the required input length of the trained models.

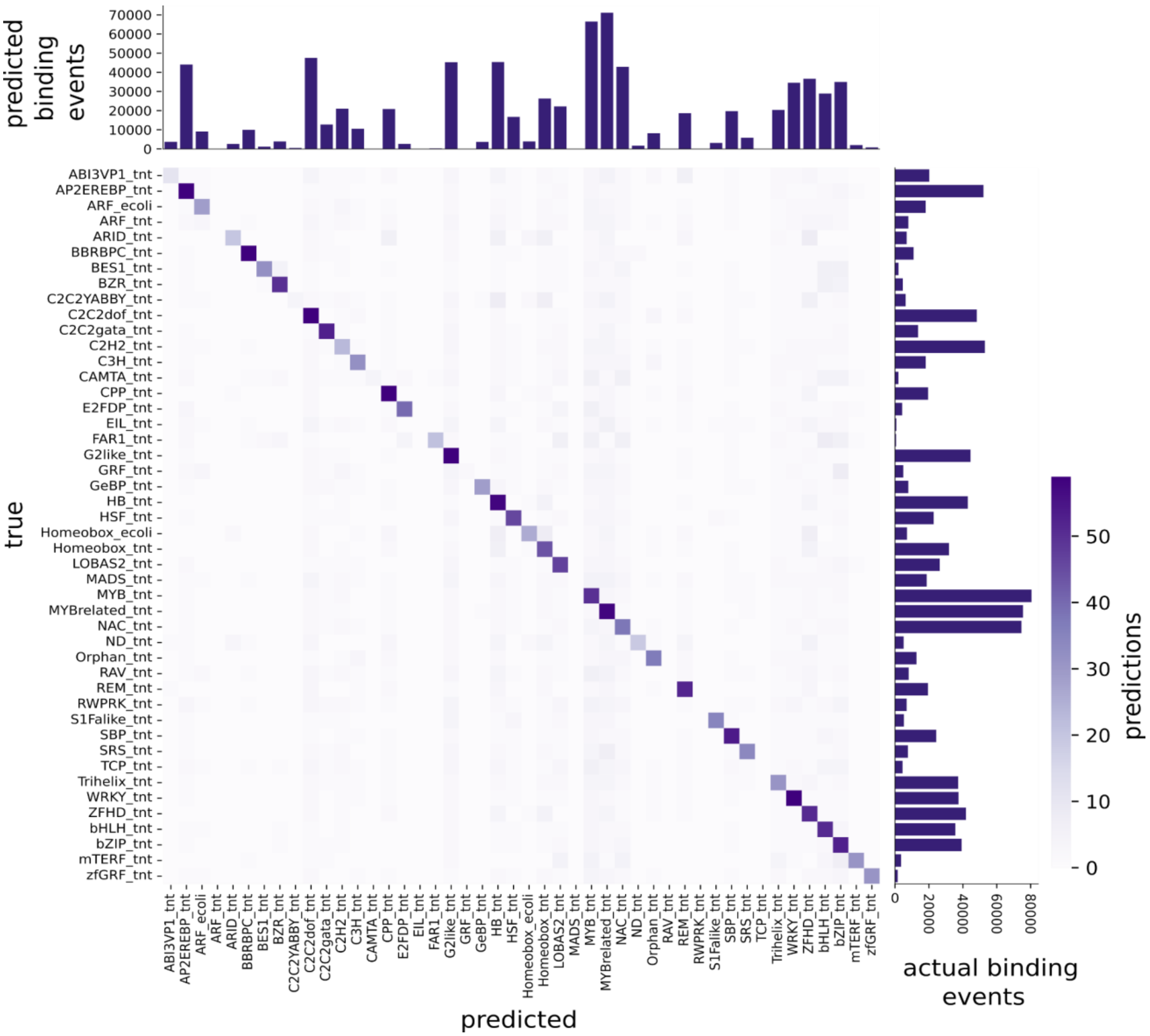

Supplementary Figure 3. The multi-label confusion matrix illustrates the predicted TF binding across the genome. The main diagonal of this matrix quantifies how well models predict the binding activity of the respective families. Additionally, the availability of experimentally verified binding events and the rate of predicted binding events are presented. Source data are provided as a Source Data file.

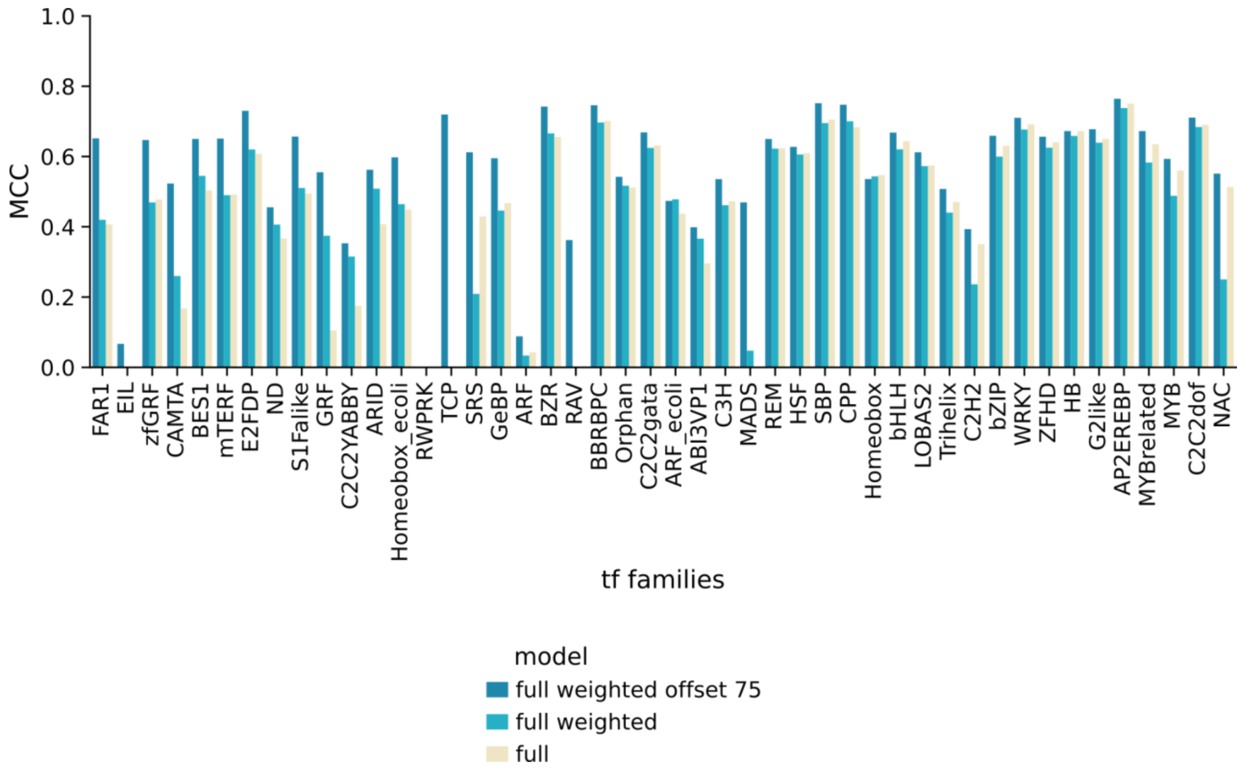

Supplementary Figure 4. Comparison of multi-label classifiers trained with different strategies to mitigate the effects of data imbalance. Compared to the unweighted model (full), the weighted (full weighted) models were trained with modified loss functions that assigned more weights to the contributions of minority classes to the total loss. In addition to weighting the loss function, the weighted full models with 75 bp offset (full weighted offset 75) upsampled the minority classes by training with 250 bp windows with 175 bp overlaps. Source data are provided as a Source Data file.

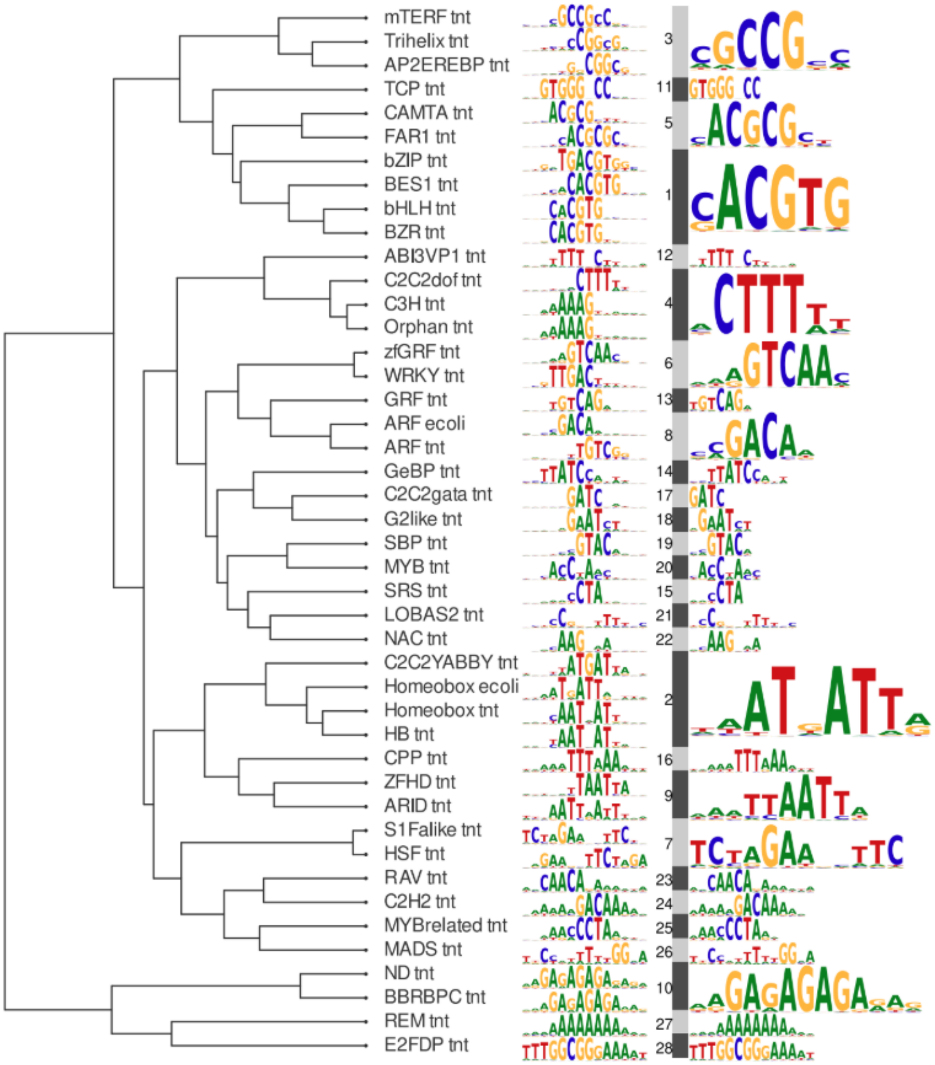

Supplementary Figure 5. Feature extraction from weighted models trained on 250 bp windows with 75 bp overlaps revealed the binding sites for other families such as the ARF_tnt, RAV_tnt and GRF_tnt which were not captured by unweighted models.

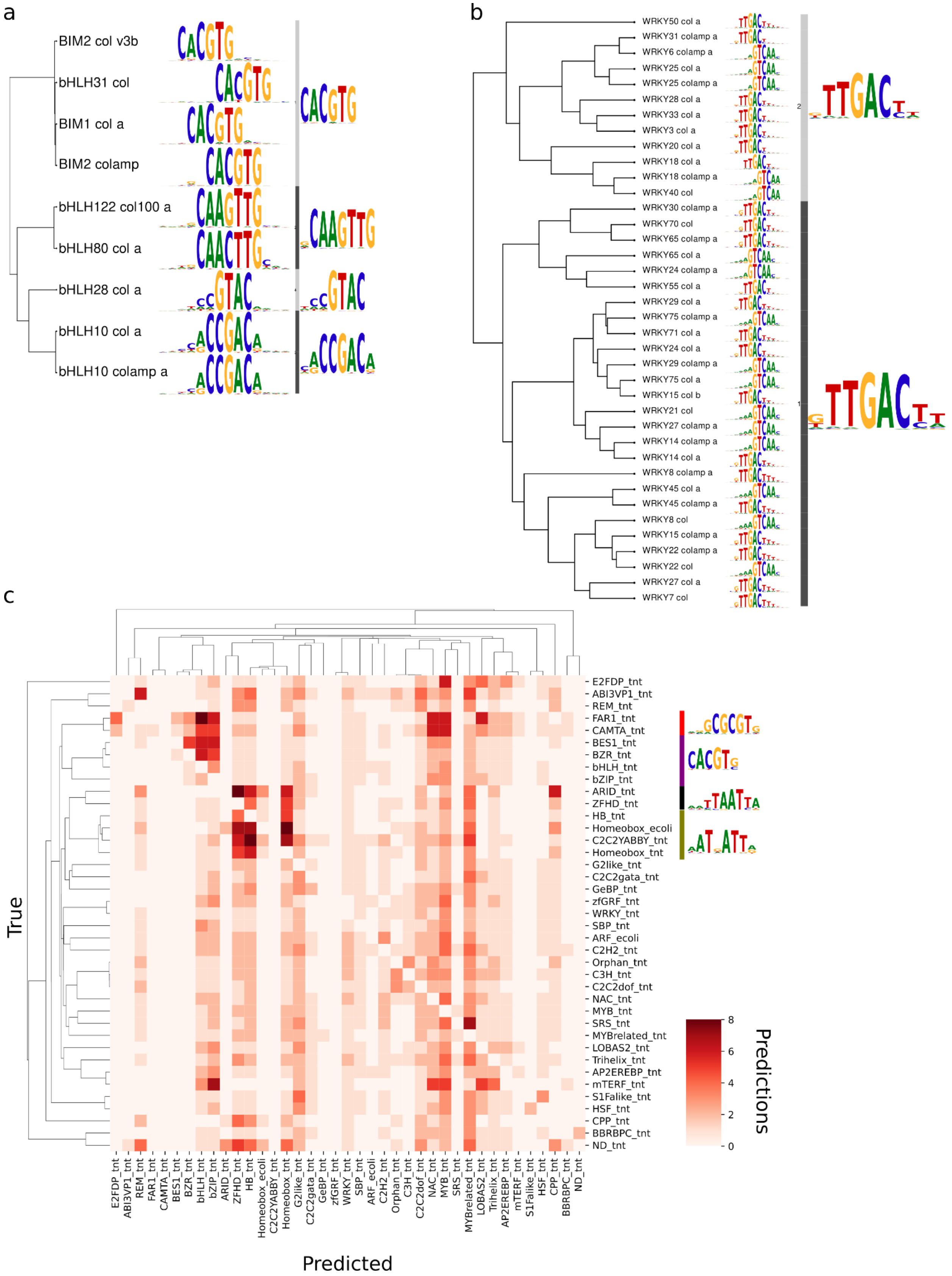
a
BIM2 col v3b
bHLH31 col
BIM1 col a
BIM2 colamp
bHLH122 col100 a
bHLH80 col a
bHLH28 col a
bHLH10 col a
bHLH10 colamp a
b
WRKY50 col a
WRKY31 colamp a
WRKY6 colamp a
WRKY25 col a
WRKY25 colamp a
WRKY28 col a
WRKY33 col a
WRKY3 col a
WRKY20 col a
WRKY18 col a
WRKY18 colamp a
WRKY40 col
WRKY30 colamp a
WRKY70 col
WRKY65 colamp a
WRKY65 col a
WRKY24 colamp a
WRKY55 col a
WRKY29 col a
WRKY75 colamp a
WRKY71 col a
WRKY24 col a
WRKY29 colamp a
WRKY75 col a
WRKY15 col b
WRKY21 col
WRKY27 colamp a
WRKY14 colamp a
WRKY14 col a
WRKY8 colamp a
WRKY45 col a
WRKY45 colamp a
WRKY8 col
WRKY15 colamp a
WRKY22 colamp a
WRKY22 col
WRKY27 col a
WRKY7 col
c
E2FDP_tnt
ABI3VP1_tnt
REM_tnt
FAR1_tnt
CAMTA_tnt
BES1_tnt
BZR_tnt
bHLH_tnt
bZIP_tnt
ARID_tnt
ZFHD_tnt
HB_tnt
Homeobox_ecoli
C2C2YABBY_tnt
Homeobox_tnt
G2like_tnt
C2C2gata_tnt
GeBP_tnt
zfGRF_tnt
WRKY_tnt
SBP_tnt
ARF_ecoli
C2H2_tnt
Orphan_tnt
C3H_tnt
C2C2dof_tnt
NAC_tnt
MYB_tnt
SRS_tnt
MYBrelated_tnt
LOBAS2_tnt
Trihelix_tnt
AP2EREBP_tnt
mTERF_tnt
S1Falike_tnt
HSF_tnt
CPP_tnt
BBRBPC_tnt
ND_tnt
True
Predicted
Predictions
0
2
4
6
8

Supplementary Figure 6. Predicted interaction predictive motifs (IPMs) for members of the WRKY and bHLH transcription factor families. a) Predicted motifs for individual members of the WRKY transcription factor family. b) Predicted motifs for individual members of the bHLH transcription factor family. c) A subsection of the diagonal masked confusion matrix highlighting false positives (columns) and false negatives (rows). Source data are provided as a Source Data file.

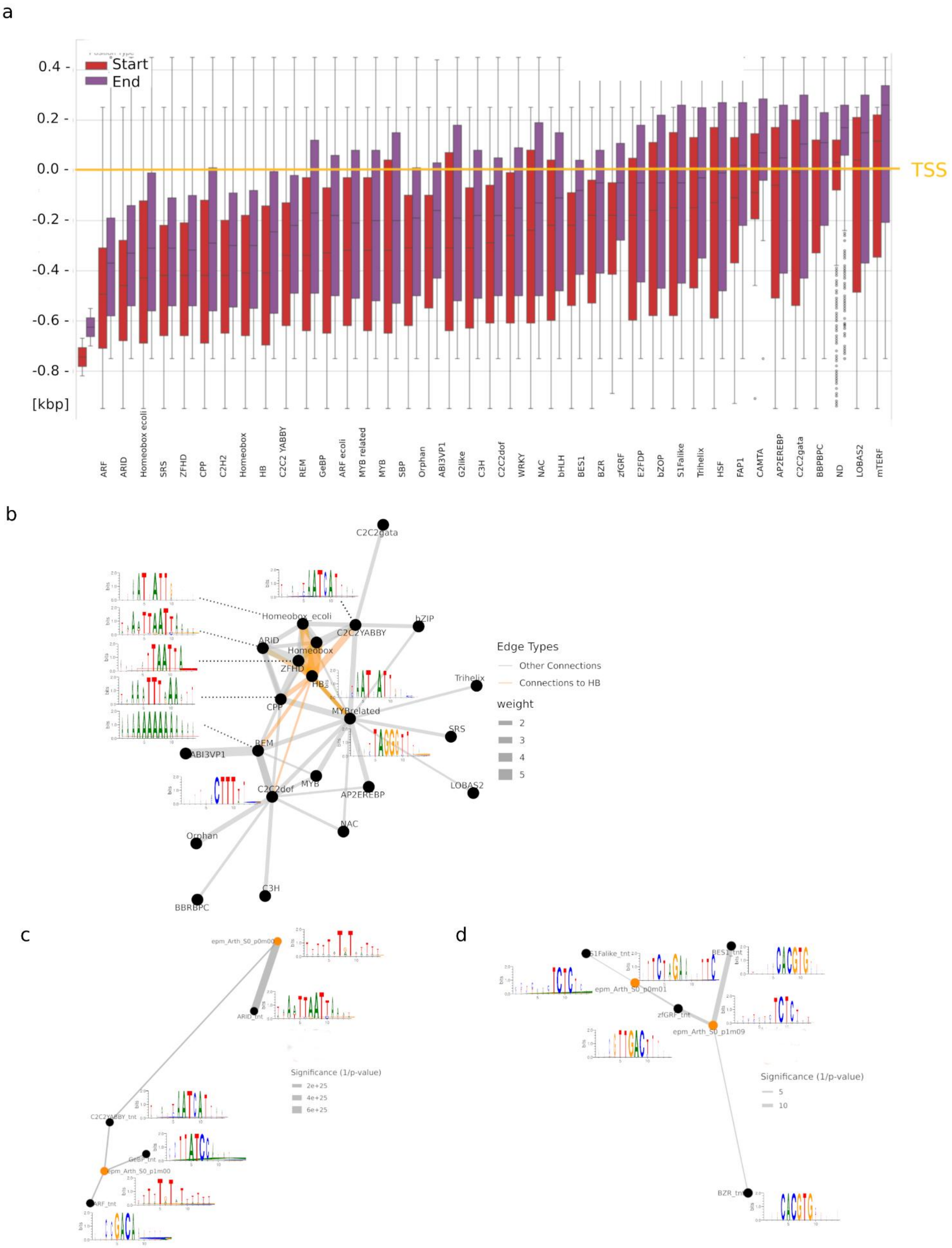
a
Start
End
0.4
0.2
0.0
-0.2
-0.4
-0.6
-0.8
[kbp]
TSS
ARF
ARID
Homeobox ecoli
SRS
ZFHD
CPP
C2H2
Homeobox
HB
C2C2 YABBY
REM
GeBP
ARF ecoli
MYB related
MYB
SBP
Orphan
ABI3VP1
G2like
C3H
C2C2dof
WRKY
NAC
bHLH
BES1
BZR
zfGRF
E2FDP
bZOP
S1Falike
Trihelix
HSF
FAP1
CAMTA
AP2EREBP
C2C2gata
BBPBPC
ND
LOBAS2
mTERF
b
C2C2gata
Homeobox_ecoli
bZIP
ARID
C2C2YABBY
Homeobox
ZFHD
HB
Trihelix
CPP
MYBrelated
SRS
REM
ABI3VP1
MYB
AP2EREBP
C2C2dof
LOBAS2
NAC
Orphan
C3H
BBRBPC
Edge Types
Other Connections
Connections to HB
weight
2
3
4
5
c
epm_Arth_S0_p0m00
ARID_tnt
Significance (1/p-value)
2e+25
4e+25
6e+25
C2C2YABBY_tnt
GeBP_tnt
epm_Arth_S0_p1m00
ARF_tnt
d
S1Falike_tnt
BES1_tnt
epm_Arth_S0_p0m01
zfGRF_tnt
epm_Arth_S0_p1m09
Significance (1/p-value)
5
10
BZR_tnt

Supplementary Figure 7. Distribution of occurrence and co-occurrence of IPMs over different genomic regions. a) Predicted binding of TF families over a rolling window (i=10) sequence windows over gene transcription start regions, with 1 kbp of upstream intergenic and 500 bp of gene body sequence with filtered derived a certainty range equivalent to a 200 bp. The box plots illustrate the upper and lower 25th percentiles, with error bars representing the minimum and maximum values, as well as the mean of the occurrences of start and end coordinates for windows in which a given TF was predicted to bind. b) Network graphs of HB TF families and co-enriched TF families are shown as central nodes (*Z*-score >=1) with weblogos of IPMs. The HB TF family is co-enriched with main TF families featuring AT-rich binding motifs and show different rates of enrichment, e.g. to the Homeobox TF family with near identical IPM. c) Enrichments for co-occurrence of IPMs and expression predictive motifs (EPMs) were calculated using a two-sided Fisher's exact test and filtered for abundance (odd-ratios >1) and significance (*p*-value >=0.05). Central nodes (orange) are EPMs that are homologous on sequence level but have different positional preferences that are linked to differential prediction of gene expression levels. The histograms show the preferred range of occurrence within gene regions (black bar) and flanking intergenic spaces (dotted bar). The sequential identical EPMs p0m00 and p1m00 occur within transcribed regions or downstream of the terminator, respectively, and are significantly co-occurring with different predicted TF-families. d) (see panel c). The sequentially identical EPMs p0m01 and p1m09 occur within transcribed regions or downstream of the terminator, respectively, and are co-occurring with different predicted TF-families. The EPMs Arth_p0m01 and p1m09 are like the binding sites of BBRBPC (BPC5) and ND. Source data are provided as a Source Data file.

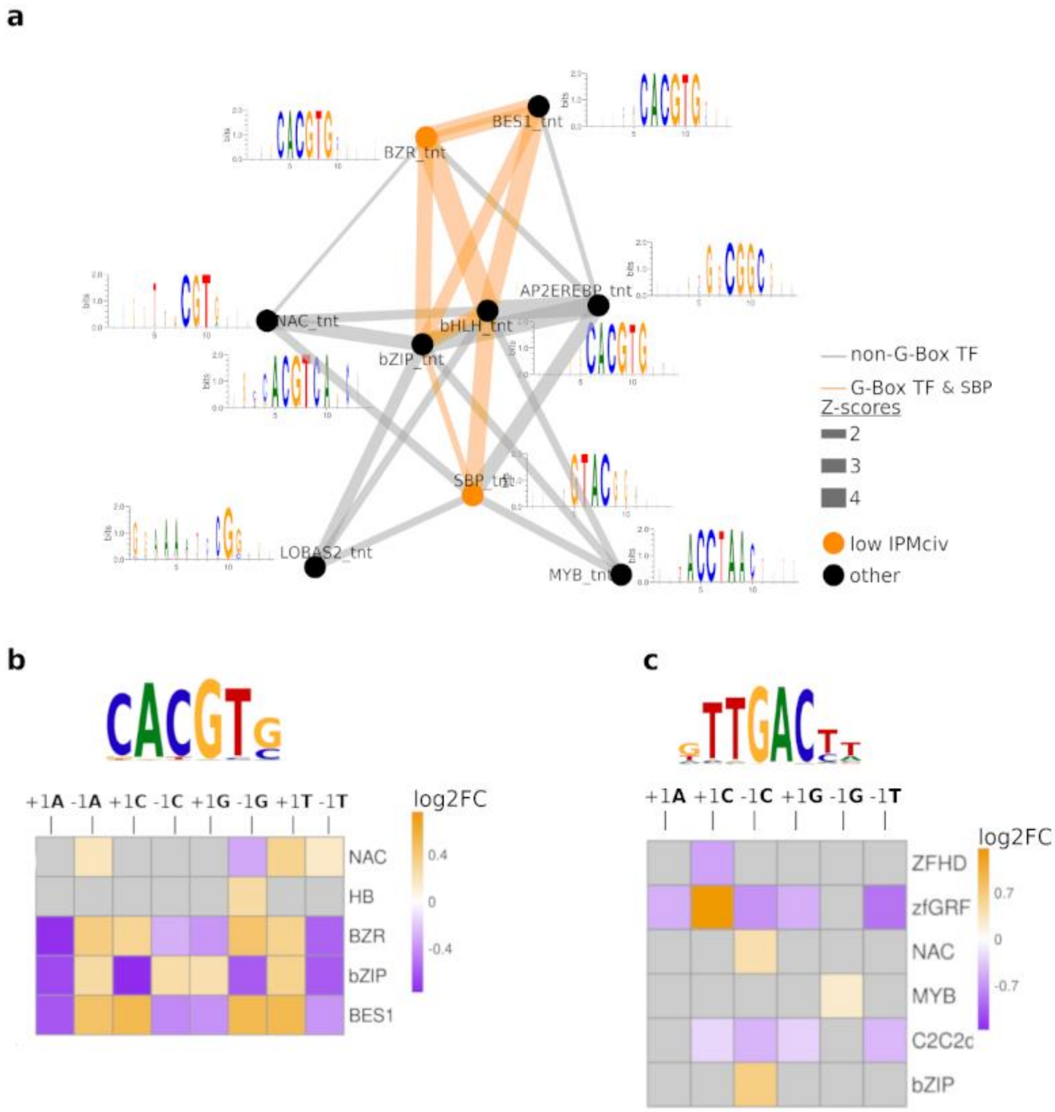

Supplementary Figure 8. Co-occurrence network of G-Box TF family and perturbation tests. a) Network graphs of G-box TF families (BZR, BES1, bHLH) and bZIP are shown as central nodes together with co-enriched TFs families (*Z*-score >=1, SBP, MYB, LOBAS2, NAC, AP2EREBP) and weblogos of IPMs. G-box TF families show different rates of enrichment and connections with other TF families despite featuring highly similar motifs. Transcription factor families BZR and SBP feature exceptionally low IPM predictability. b) Variants were introduced to the +1 and -1 bp positions of the G-box motif 5'-CACGTG-3' (n = 1000) to control perturbations. The log2 fold change (log2FC) in co-occurrences of other TF families without a loss of (BZR, BES1, bHLH and bZIP) was measured, and data was filtered by two-sided Fisher's exact test *p*-value > 0.01. c) Variants were introduced to the +1 and -1 bp positions of the WRKY motif 5'-TTGAC-3' (n = 1000) to control perturbations. The log2 fold change (log2FC) in co-occurrences of other TF families without a loss of WRKY was measured, and data was filtered by a *p*-value > 0.01 (two-sided Fisher's exact test).

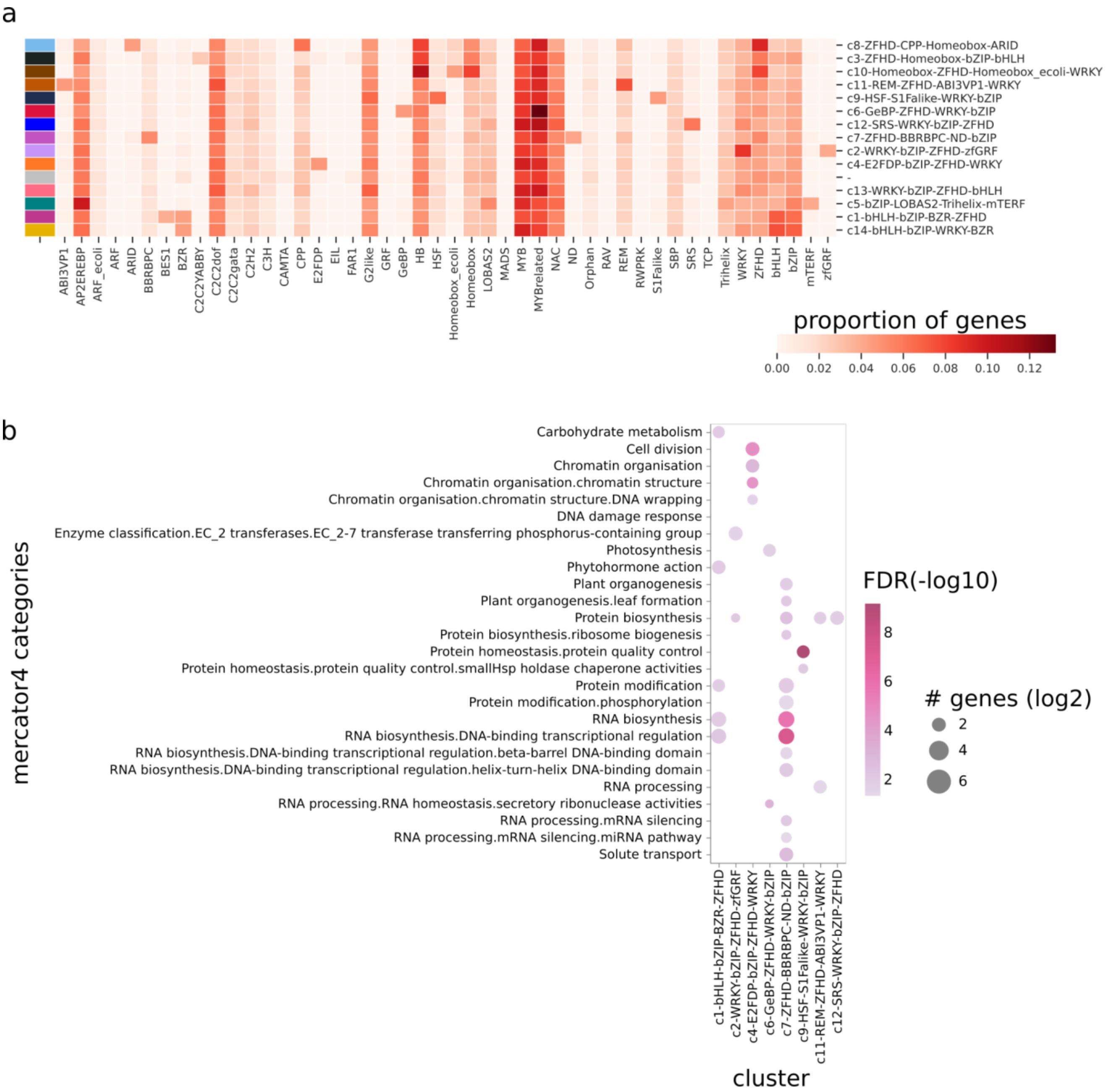

Supplementary Figure 9. Clustering of genes based on predicted regulatory profiles. A heatmap showing enrichment transcription factor families across 14 regulatory clusters. Large families such as MYB, MYBrelated and AP2EREBP are differentially enriched across clusters while smaller families such as the ARID, mTERF and zGRF are cluster specific. b) Enrichment of regulatory clusters in Mercator4 functional categories (two-sided Fisher's exact test; FDR ≤ 0.05). Source data are provided as a Source Data file.

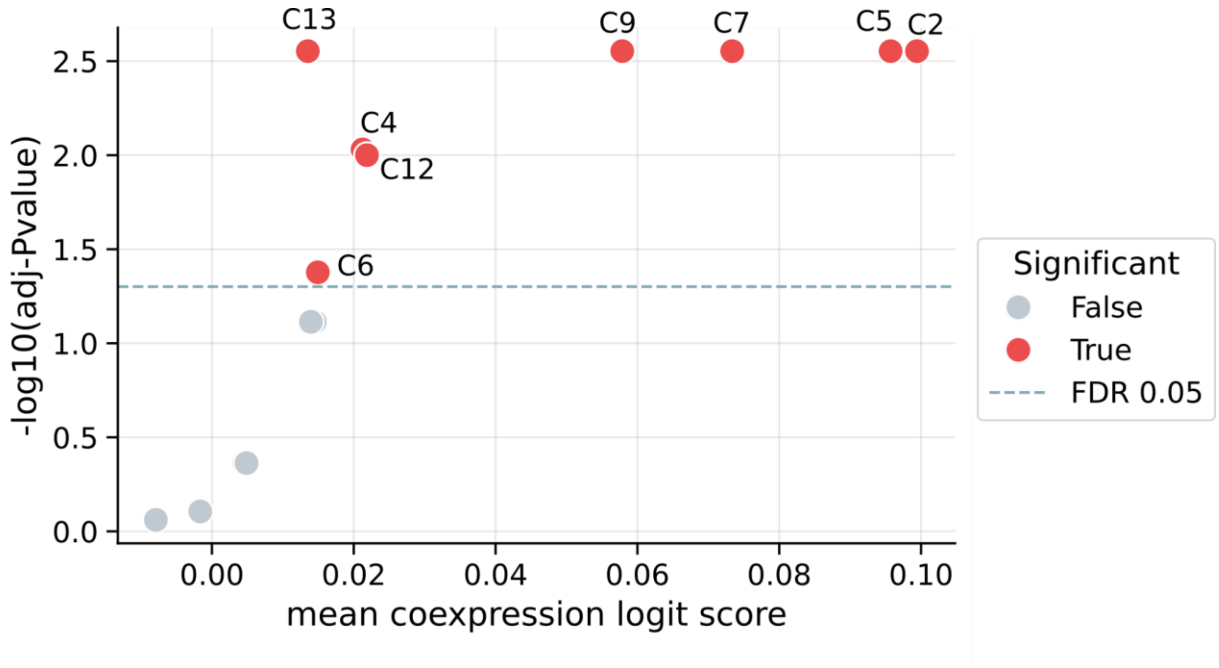

Supplementary Figure 10. Permutation test (n=1000) revealed that genes belonging to the same regulatory binding cluster are co-expressed. Genes belonging to eight of the fourteen regulatory clusters show significantly higher co-expression logit scores compared to genes selected randomly from the pool of fourteen clusters. Source data are provided as a Source Data file.

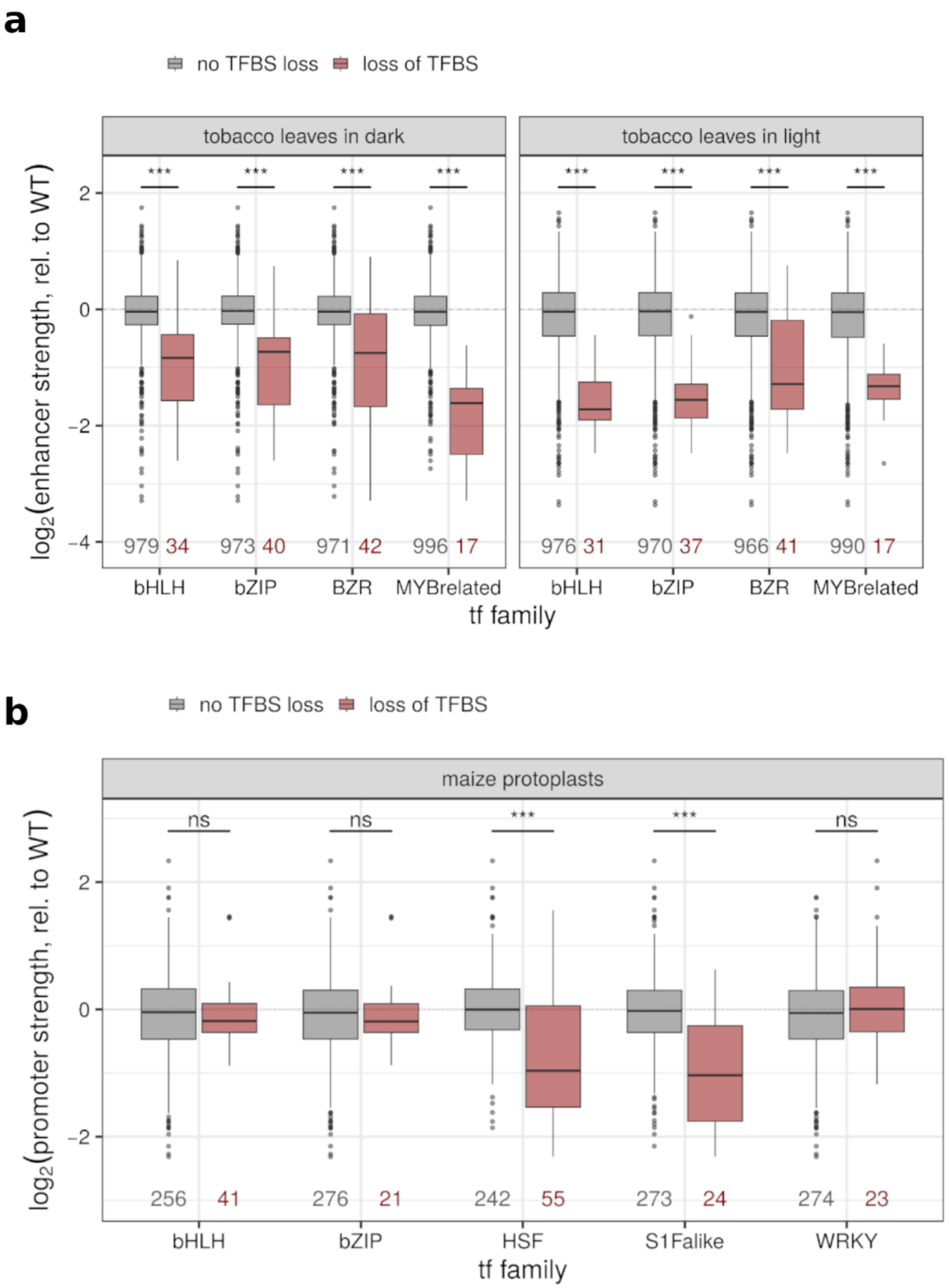

Supplementary Figure 11. Predicted dynamics in TF family binding and changes in gene expression measured in plant STARRseq. a) Transcription factor binding was predicted for all possible single-nucleotide substitution variants of the rbcS-E9 enhancer. The enhancer strength as measured by Jores *et al.* [2] in tobacco leaves in normal light dark cycles (light) or in complete darkness (dark) of the variants relative

to the wildtype (WT) enhancer is plotted for variants with (grey) or without (red) a loss of binding sites for the indicated transcription factor family. Boxplots display the median, with upper and lower 25th percentiles indicated by whiskers and outliers as individual dots. The Bonferroni-adjusted *p*-value from a two-sided Wilcoxon rank-sum test is indicated. : *, $p \leq 0.05$; **, $p \leq 0.01$; ***, $p \leq 0.001$; ns, not significant. b) Transcription factor binding was predicted for two to three single- or double-nucleotide substitution variants of approximately 100 plant core promoters. The promoter strength as measured by Jores *et al.* [3] in maize protoplasts of the variants relative to the corresponding wildtype (WT) promoter is plotted for variants with (grey) or without (red) a loss of binding sites for the indicated transcription factor family. Boxplots display the median, with upper and lower 25th percentiles indicated by whiskers and outliers as individual dots. The Bonferroni-adjusted *p*-value from a 2-sided Wilcoxon rank-sum test is indicated: *, $p \leq 0.05$; **, $p \leq 0.01$; ***, $p \leq 0.001$; ns, not significant. Source data are provided as a Source Data file.

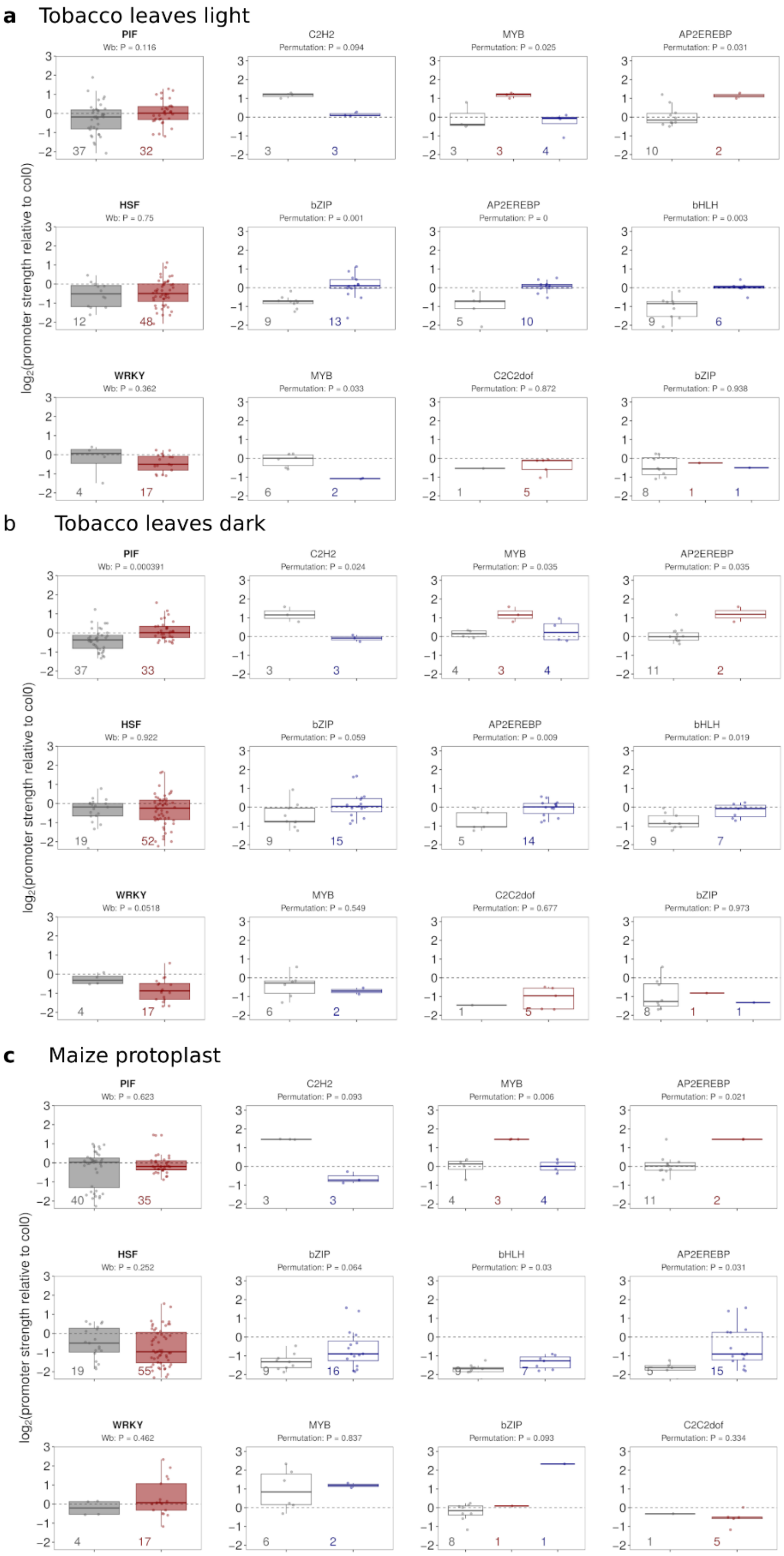
a Tobacco leaves light
PIF
Wb: P = 0.116
C2H2
Permutation: P = 0.094
MYB
Permutation: P = 0.025
AP2EREBP
Permutation: P = 0.031
HSF
Wb: P = 0.75
bZIP
Permutation: P = 0.001
AP2EREBP
Permutation: P = 0
bHLH
Permutation: P = 0.003
WRKY
Wb: P = 0.362
MYB
Permutation: P = 0.033
C2C2dof
Permutation: P = 0.872
bZIP
Permutation: P = 0.938
log2(promoter strength relative to col0)
b Tobacco leaves dark
PIF
Wb: P = 0.000391
C2H2
Permutation: P = 0.024
MYB
Permutation: P = 0.035
AP2EREBP
Permutation: P = 0.035
HSF
Wb: P = 0.922
bZIP
Permutation: P = 0.059
AP2EREBP
Permutation: P = 0.009
bHLH
Permutation: P = 0.019
WRKY
Wb: P = 0.0518
MYB
Permutation: P = 0.549
C2C2dof
Permutation: P = 0.677
bZIP
Permutation: P = 0.973
c Maize protoplast
PIF
Wb: P = 0.623
C2H2
Permutation: P = 0.093
MYB
Permutation: P = 0.006
AP2EREBP
Permutation: P = 0.021
HSF
Wb: P = 0.252
bZIP
Permutation: P = 0.064
bHLH
Permutation: P = 0.03
AP2EREBP
Permutation: P = 0.031
WRKY
Wb: P = 0.462
MYB
Permutation: P = 0.837
bZIP
Permutation: P = 0.093
C2C2dof
Permutation: P = 0.334

Supplementary Figure 12. Changes in gene expression measured in plant STARRseq comparing retained and dynamics in TF binding after introduction of variation. a) Changes in gene expression for TF PIF (bHLH family), HSF and WRKY in tobacco light conditions, were not significant comparing retained and loss of binding using Wilcoxon rank sum test (filled box plots), while permutation test of co-retained (grey), co-loss (red) or co-gained (blue) TF bindings show significant changes (empty box plots). Boxplots display the median, with upper and lower 25th percentiles indicated by whiskers and outliers as individual dots. Positive or negative changes in measured transcript levels highlight differences between control and variant samples. b) Changes in gene expression for TF PIF (bHLH family), HSF, and WRKY in tobacco dark conditions. See text of panel a) for further information. c) Changes in gene expression for TF PIF (bHLH family), HSF, and WRKY in maize protoplast conditions. See text of panel a) for further information.

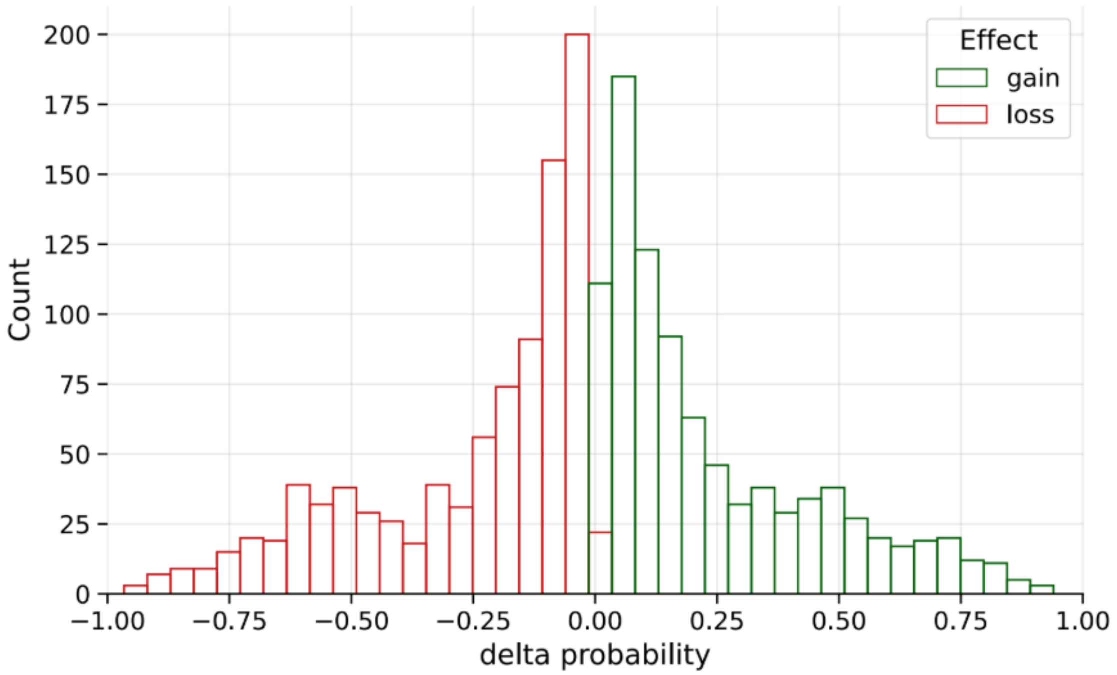

Supplementary Figure 13. The distribution of predicted probability changes (Δ prob) for 250 bp windows where an SNP led to a predicted gain or loss in TF binding.

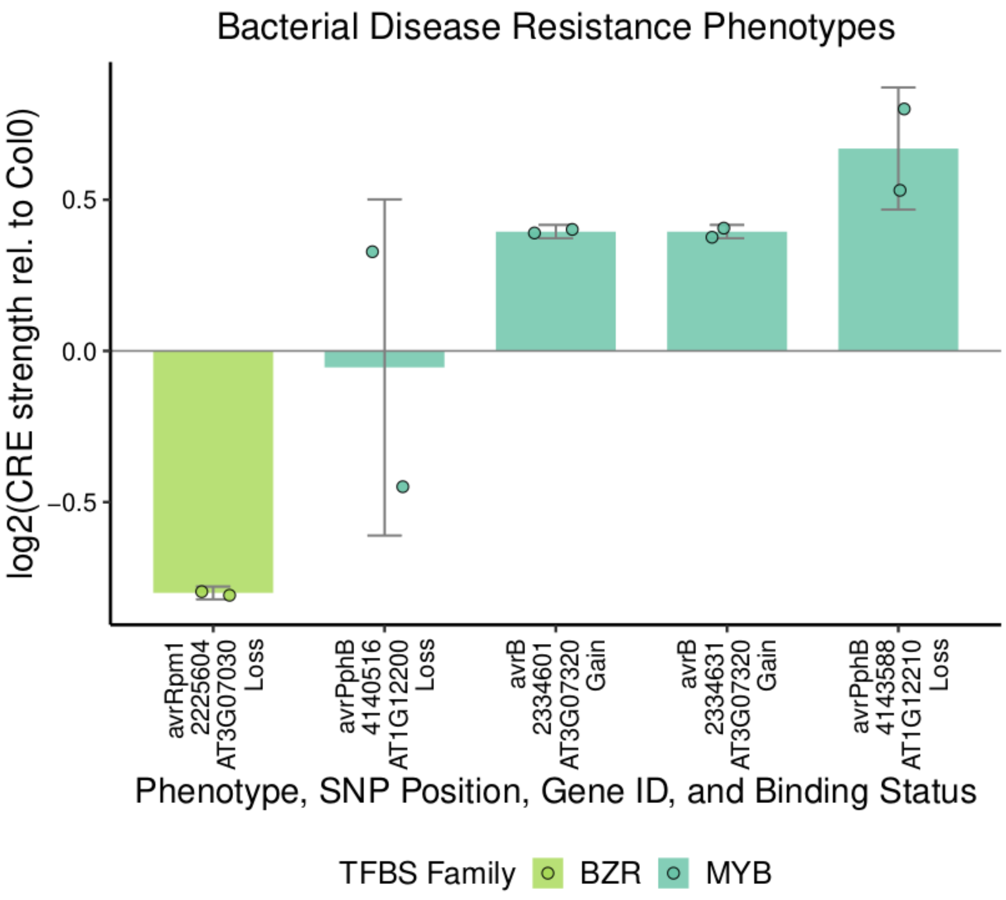

Supplementary Figure 14. Changes in relative promoter strength following predicted gain and loss of TF binding for GWAS variant constructs associated with the bacterial disease resistance phenotype, as measured by plant STARR-seq. The data is presented as the mean log2 relative strength of the variant compared to the Col0 baseline across n=2 paired biological replicates from the high-throughput plant STARR-seq screening assay. Error bars represent standard deviation, with individual replicate pairs overlaid as distinct data points. Source data are provided as a Source Data file.

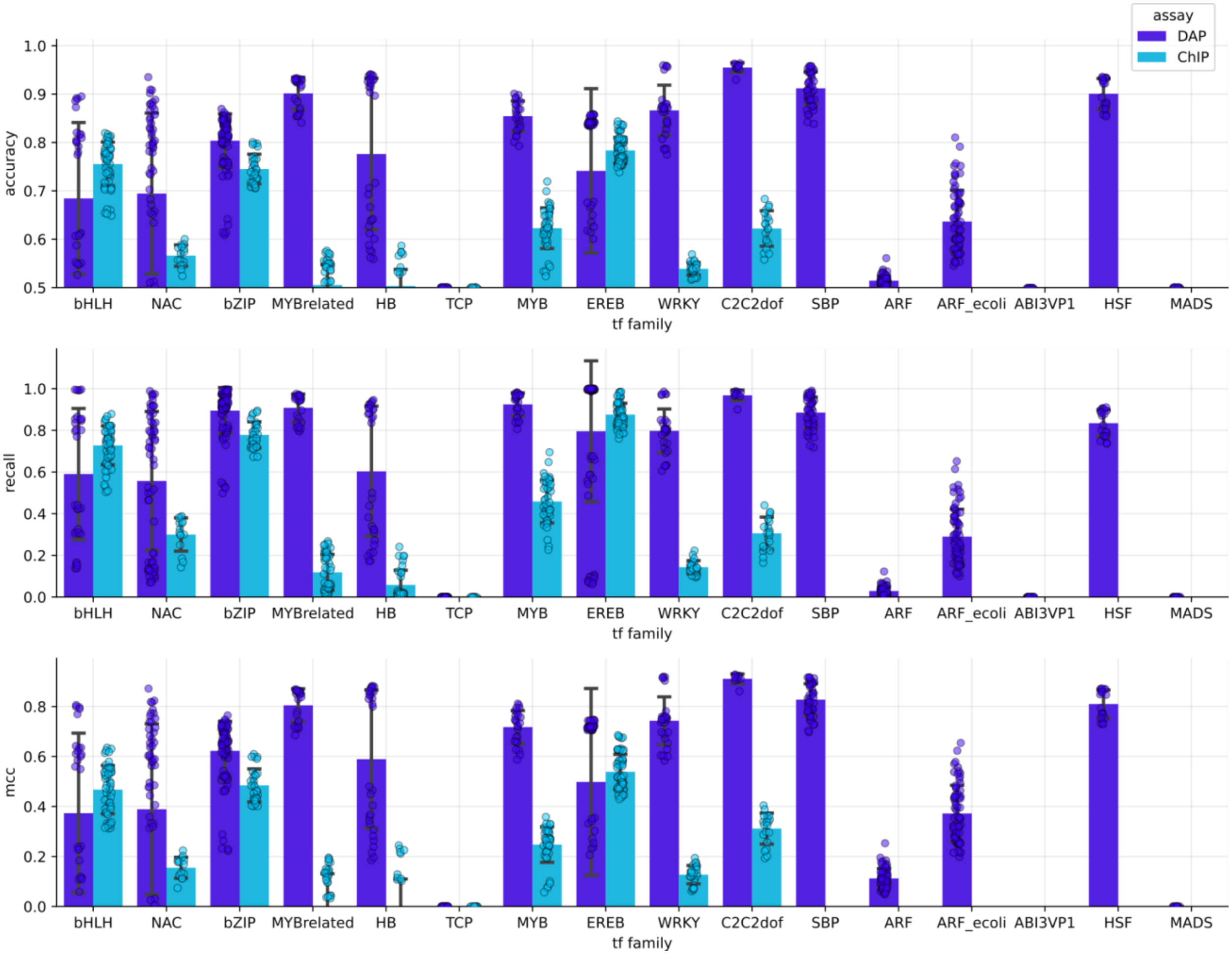

Supplementary Figure 15. Model performance of multi-label classifiers on DAP-seq and ChIP-seq datasets for *Zea mays*. Bar plots show the mean performance across ChIP- and DAP-seq datasets, with error bars indicating standard deviation. Multi-label classifiers demonstrate the potential for transfer of knowledge between species while also highlighting the challenges posed by latent factors influencing *in vivo* DNA binding. Source data are provided as a Source Data file.

Supplementary Note 1. Model performance across gene proximity regions

To further evaluate the performance of our models in identifying putative CREs in the proximity of their nearest gene, we extracted 4500 bp around the transcription start site (TSS) and transcription termination site (TTS) of *A. thaliana* genes (including 500 bp of the transcribed region either site). The 4500 bp fragments were tiled into non-overlapping 250 bp windows to fit the expected input sizes of our models, allowing us to evaluate the trained models on the 250 bp tiles (Supplementary Fig. 2). The distribution of the predicted binding events indicates that the majority of the correctly predicted binding events are located around the TSS within the promoter regions. In contrast, the lowest proportions of correctly predicted binding events were observed in the windows just before the TTS, harbouring the 3' UTRs and downstream coding sequences.

Supplementary Note 2. Weak predictive performance for low data abundance TF family

In the ARF transcription factor family, approximately 61,000 homologous sites were identified on chromosome 1, with only 6% of matches being correctly predicted of 11,654 associated DAP-seq targets (Supplementary Data 2 and 4). The low sensitivity (0,3 %) indicates that most errors were due to false negatives, resulting from low importance of the respective IPM (Supplementary Fig. 4). The poor performance for ARF predictions can likely be attributed to the small number of ARF binding sites available in the DAP-seq dataset (Supplementary Fig. 1). Although the ARF motif is prevalent across chromosome 1, the limited training data made it challenging to learn meaningful sequence context for accurate predictions. Supporting this, O'Malley and colleagues reported that only about 30% of experimentally observed peaks contained ARF binding sites, which aligns with the sensitivity observed in [1] (Supplementary Fig. 4).

Supplementary Note 3. TF families are not equally distributed in promoter regions

Using the multi-label classification model to predict on sequences upstream of gene protomer regions, we could confirm that distinct TF families are more likely to co-occur than others based on their genomic location (Supplementary figure 5). LOBAS2, with a median positioning 221 to 11 bp upstream of the TSS, overlaps with AP2/EREBP positioning approximately 242 to 51 bp (Supplementary figure 5a). Despite overlapping occurrences upstream of the TSS, with HB occurring 433 to 227 bp upstream of the TSS, AP2/EREBP and LOBAS2 demonstrate enriched co-occurrence (*Z*-score 3.68), which contrasts with the co-occurrence of AP2/EREBP and HB (*Z*-score 0.27) (Figure 4f), supporting a non-random distribution and recognition of the TFBS by the model.

We also identified HB-box TF families as an outlier with low IPMciv but high sensitivity (Fig. 3a), binding to 5'-SAATHATTS-3'. IPMs of the HB-box TF families are co-occurring with MYBrelated, homeobox and ZFHD motifs (Supplementary figure 5b). Among these, the IPM of HB and homeobox are near identical (5'-SAATHATTS-3'). For homeobox, predicted bindings appear to be independent of the measured DAP-seq interactions and are much smaller with only 603 occurrences of the cognate IPM (Supplementary Data 4). This indicates that the prediction of HB, and Homeobox, is fully dependent on sequence context, too, which is likely contributed by the occurrence of ZFHD, MYBrelated and others.

In addition to co-enrichment with other TF families, we hypothesized that the model's binding predictions could distinguish functional gene regions such as promoters, UTRs, and terminators. Thus, we analysed the co-occurrence of interaction predictive motifs (IPMs) with expression predictive motifs (EPMs), sequence features associated with prediction of the gene expression level [4]. For example, epmArth-S0-p1m00 is enriched in the terminator region and is co-enriched with TFs like C2C2-YABBY, GeBP, and ARF, while 5' promoter regions were enriched for epmArth-S0-p1m00 alongside ARID and C2C2-YABBY (Supplementary Fig. 5c, Supplementary Data 5). Interestingly, we previously observed that epmArth-S0-p0m01 is associated with high gene expression in leaves under standard conditions in 95% of the transcripts where it is present [4]. This EPM is similar to 5'GAGA'3 situated in the 5'UTR of genes and is co-enriched with S1Falike or zfGRf (Supplementary Fig. 5d). These results suggest that protein-DNA interaction models can effectively distinguish between gene features, including promoter, terminator, and UTR regions, likely defined by distinct combinations of CREs.

Supplementary Note 4. TFBS sequence flanking perturbations change combinatorial binding prediction

To explore the importance of sequences flanking the core motif, we introduced single nucleotide mutations adjacent to canonical TFBS and analysed their impact on the predicted co-occurrence of other binding sites. For bHLH, shifts of +1/-1 positions significantly modified predicted TFBS co-occurrence with other G-box binding families (bHLH, BZR, bZIP, BES1) in a sequence- and position-dependent manner (Supplementary Fig. 6). *In silico* mutations of the AP2/EREBP core motif, resulted in decrease of co-occurring factors of bHLH, LOBAS2, mTERF, MYB and Trihelix. Similarly, mutations adjacent to WRKY, specifically +1T and +1C, substantially influenced predictions. The +1T mutation adversely affected WRKY co-occurrence with C2C2dof, HSF, MYBrelated, and NAC, while +1C augmented WRKY and zfGRF co-occurrence. These observations underscore two principal insights: the association between WRKY and zfGRF exhibits high sensitivity to partially overlapping TFBS, consistent with entries in the JASPAR database. Secondly,

WRKY and HSF/NAC associations demonstrate that mutations outside consensus, non-overlapping TFBS can still modify predicted co-occurrence, even in the absence of binding site alteration.

This implies that TFBS combinations incorporate an expanded sequence code beyond core motifs, potentially encompassing multiple partially overlapping and non-overlapping TFBS. This proposition is substantiated by G-Box binding factors (bZIP, BZR, bHLH, BES1), where co-occurrence ratios are influenced by the +1/-1 position relative to the core CACGTG motif. Most interestingly, complementary symmetric mutations (-1A/+1T, -1G/+1C, -1T/+1A) yield comparable effects, emphasizing the extended palindromic nature of the G-Box. These findings underscore the importance of sequence context and motif combinations in G-box-related binding predictions, as overall, other high performing TF families appear to be co-enriched with one to multiple partners like AP2/EREBP, LOBAS2 and HB, while others like BBRBPC is rather exceptional, where high sensitivity results from a unique and important IPM (Supplementary Fig. 6).

Supplementary Note 5. Specific TFBS have distinct patterns of predicted co-occurrences

Interestingly, the perturbations could be found to be reflected in the predicted co–occurrence of specific TF family members. Individual members of the bHLH family characterized by ChIP-seq (n= 8), DAP-seq (n= 10) and PBM (n= 33) could be recovered at rates of 77.3%, 78.3% and 93.6%. In contrast, the model showed lower recovery rates for BZR and BES1 binding at these same sites on average 50.9% and 24.7%, indicating false positive prediction and/or combinatory binding. Interestingly, recovery rates for characterised TFBS and their combinations could vary strongly. While for example all sites of bHLH104 were recovered by the bHLH model, the model predicted binding in 30.2%, 22.9% and 71.8% of the sites for BES1, bZIP and BZR, respectively too. In the case of bHLH13 99.9% of sites were recovered by bHLH predictions, yet 53,5%, 87.7% and 32,2% were predicted to be bound by BES1, bZIP and BZR, too (Supplementary Data 4). This observation further indicates the dependency of the model's prediction on sequence context for combinatory predictions and highlights its relevance in the in-detail analysis of potential regulatory regions.

Supplementary Note 6. Statistical evaluation of variant effect prediction on AraGWAS

To demonstrate the model's applicability, we obtained data for SNPs in *A. thaliana* from the AraGWAS catalogue that were significantly associated with phenotypes in a number of GWAS [5,6]. We filtered these and retained a total of 7364 SNPs found within 1500 bp flanking regions of genes (1000 bp and 500 bp outside and inside the transcribed gene region, respectively), out of which 54.5% were in promoter (upstream) and 45.5% were in terminator (terminator) regions. We observed perturbations in binding

prediction due to the filtered SNPs and noticed that 20.72% of those SNPs led to either a gain or loss in predicted binding sites (Fig. 6a).

To assess the significance of this result for each SNP, 30 *in silico* SNPs were generated with similar nucleotide substitutions at distinct random positions within the prediction window. For example, if the SNP was a mutation from C to A, we generated 30 C to A nucleotide substitutions at other randomly selected positions and recorded the perturbation effect independently. The change of predicted binding following the introduction of *in silico* SNPs was classified into three cases, where random mutations led to (1) an expected perturbation (identical to the observed perturbation with the SNP), (2) a *de novo* perturbation, or (3) no perturbation. For every SNP that changed predicted binding, more than half of the *in silico* SNPs resulted in no perturbation, about 13% of cases resulted in the expected perturbation, and about 33% of cases resulted in a *de novo* perturbation (Fig. 6b). To ensure that the predicted gains and losses in transcription factor (TF) binding were not artifacts of minor statistical fluctuations around the classification threshold (e.g., marginal shifts between 0.49 and 0.51), we systematically quantified the change in predicted probability for all variants. The resulting perturbations were far from marginal, as 41.73% of the binding changes exhibited a robust, high-confidence shift in probability larger than 0.2 (Supplementary Fig. 13).

Supplementary Note 7. Changes in gene expression for the phenotype bacterial disease resistance from predicted variant effects on TF binding

We predicted a loss of MYB by SNP on chromosome 1 position 4143588, congruently associated with bacterial disease resistance likely affecting regulation of the AT1G12210 (RFL1) gene similar to adjacent functionally studied and extensively reviewed bacterial disease resistance gene RPS5 [7] (Supplementary Fig. 9). In addition, we detected larger changes in gene regulation after predicted loss of BZR and gain of MYB likely affecting AT3G07030 (chr. 3 position 2225604) and AT3G07320 (chr. 3 position 2334601 and 2334631), respectively associated to bacterial disease resistance. The gene AT3G07030 (ALBA6) is involved in the separation of stress granules and chaperoning HSF mRNA mediating thermotolerance [8]. The gene AT3G07320 encodes a O-Glycosyl hydrolases family 17 protein.

Supplementary Note 8. Application of multi-label classifiers for cross-species peak annotation

An advantage of our multi-label classifiers to model DNA-TF interaction is their application as peak annotation tools across different species. MOA-seq provides a scalable and affordable approach to capture genome-wide protein binding events, primarily only restricted to accessible chromatin [9]. Since MOA-seq is a recent technique, ATAC-seq data might be more widely available in some cases or faster to generate

due to its straightforward protocol without library preparation. Both methods show a strong overlap, with MOA-seq recovering 76% and 92% of ATAC-seq peaks in Maize and Arabidopsis, respectively, and ATAC-seq recovering 35% and 71% of the MOA-seq peaks [9,10]. The higher number of peaks/ TF footprints found with MOA-seq is likely caused by the smaller size of the MNase, allowing detection of sites not accessible to Tn5. In maize, MOA-seq peaks widely overlap with TF binding sites determined by ChIP-seq (66%) even when comparing different tissues [9]. Due to the higher number of TF footprints and the higher resolutions of MOA-seq compared to ATAC-seq (thanks to the exonuclease activity of MNase), we opted for this technique as a more comprehensive set for comparison.

Supplementary Methods 1. The TF-family offset score

The offset independence score (Soi) for each predicted TF family was calculated as the ratio of continuous positive predictions within this sliding window framework. It is formally defined as:

$$Soi = \frac{Wc}{24} \qquad \textbf{(1)}$$

Here window count (Wc) is the number of consecutive overlapping windows in which the TFBS was positively predicted. The offset independence score ranges from 1, indicating a prediction that is independent of its position within the window and can be localized to a 10 bp region anywhere in the prediction window, to a minimum of 0.042, indicating a prediction highly contingent on the full 250 bp sequence context and centre placement of the motif for detection. From the offset independence score, we derived a certainty range (Rc), which estimates the effective sequence length required for a prediction:

$$Rc = 250 - (240 \times Soi) \qquad \textbf{(2)}$$

This metric was then used to filter TFBS predictions with offset independent score larger than 0.2034 equivalent to a 200 bp window around the TSS, to refine the prediction and localization of binding events, exemplarily. We assessed whether certain IPMs that were extracted from the *A. thaliana* DAP-seq multi-label classification model (like ARF-related motifs) were more enriched in specific genomic regions and predicted positively than would be expected by chance. This analysis was performed using standard functions and customs scripts in phyton using pandas, matplotlib and numpy libraries.

Supplementary Methods 2. The TF-family co-occurrence

The weighted averaged co-occurrence stands for the number of distinct TF families that are co-predicted in windows where a specific TF family of interest is present. The score was calculated as follows:

$$TFocc(k) = \frac{\sum_{i=1}^{N} \ (Bi \times Pik)}{\sum_{i=1}^{N} \ Pik} \qquad \textbf{(3)}$$

To quantify the co-occurrence tendency for each transcription factor family, a weighted average co-occurrence score (TFocc) for the specific TF family of interest (k) is calculated. This metric is the mean number of distinct TF families predicted to bind in any given 250 bp window (i) where a specific TF family of interest (TFocc) is also present. The calculation begins by summing the total number of predicted TF families binding events (Bi) across all windows, but only for the subset of windows where the specific factor of interest is present. This sum, which is the total co-occurrence count for TFocc, is then normalized by dividing it by the total number of windows in which TFocc was predicted. The resulting quotient provides a single, quantitative measure of the average combinatorial complexity of the binding environment for each specific TF family. This analysis was performed using standard functions and customs scripts in phyton using pandas, matplotlib and numpy libraries.

Supplementary Methods 3. The Interaction Predictive Motif (IPM) context importance and predictability

To evaluate the importance for predictions of IPMs alone and their context, we developed the IPM context importance value (IPMciv) (χ), where the total number of IPM occurrences that are predicted for binding of the corresponding TF family (α) is divided by the total number of IPM occurrences of the cognate TF family (b):

$$\chi = 1 - \left(\frac{\alpha}{b}\right) \qquad \textbf{(4)}$$

The predictability (P) of each IPM can be calculated as the result of subtracting 1 from the reciprocal of the IPMciv (χ):

$$P = \left(\frac{1}{\chi}\right) - 1 \qquad \textbf{(5)}$$

In addition, we calculated a score to estimate individual false positives and false negatives (y) rates dividing the IPM occurrences that are predicted for binding of the cognate TF family (α) by the experimentally measured binding events (c). If y is smaller or larger than 1, discrepancies in the model's predictive performance can be explained by the prevalence of false negatives or false positives, respectively:

$y = \frac{\alpha}{c}$

To assess the sensitivity of our models to TF co-occurrences, *in silico* mutations were introduced next to canonical TFBS sites on *A. thaliana* chromosome 1 identified by the mapping of IPM.

Supplementary Methods 4. The Interaction Predictive Motif (IPM) flanking region perturbation test

We targeted 500 sites each for bHLH (5'-CACGTG-3'), and WRKY (5'-GTCAA-3'), converting the +1 and -1 bp positions adjacent to the core binding motifs to A, C, G, or T without changing the core motif. We then predicted TF binding for these systematically modified 250 bp windows and compared the resulting changes in TFBS co-occurrence to the original predictions, while the prediction for the targeted site was still retained. The gain or loss dynamics of TFBS other than AP2EREBP, bHLH, and WRKY, respectively, were measured and tested for significance using two-sided Fisher's exact test (Supplementary Fig. 6). Further, we tested for positive enrichments of IPMs and EPMs using custom R scripts calculating *Z*-scores and two-sided Fisher's exact test (Supplementary Data 5, Supplementary Fig. 5).

Supplementary references

1. O'Malley, R. C. *et al.* Cistrome and epicistrome features shape the regulatory DNA landscape. *Cell* **165**, 1280–1292 (2016).
2. Jores, T. *et al.* Small DNA elements can act as both insulators and silencers in plants. *Plant Cell* **37**, koaf084 (2025).
3. Jores, T. *et al.* Synthetic promoter designs enabled by a comprehensive analysis of plant core promoters. *Nat. Plants* **7**, 842–855 (2021).
4. Peleke, F. F., Zumkeller, S. M., Gültas, M., Schmitt, A. & Szymański, J. Deep learning the cis-regulatory code for gene expression in selected model plants. *Nat. Commun.* **15**, 3488 (2024).
5. Togninalli, M. *et al.* AraPheno and the AraGWAS Catalog 2020: a major database update including RNA-Seq and knockout mutation data for *Arabidopsis thaliana*. *Nucleic Acids Res.* **48**, D1063–D1068 (2020).
6. Togninalli, M. *et al.* The AraGWAS Catalog: a curated and standardized *Arabidopsis thaliana* GWAS catalog. *Nucleic Acids Res.* **46**, D1150–D1156 (2018).
7. Pottinger, S. E. & Innes, R. W. RPS5-mediated disease resistance: Fundamental insights and translational applications. *Annu. Rev. Phytopathol.* **58**, 139–160 (2020).
8. Tong, J. *et al.* ALBA proteins confer thermotolerance through stabilizing HSF messenger RNAs in cytoplasmic granules. *Nat. Plants* **8**, 778–791 (2022).
9. Savadel, S. D. *et al.* The native cistrome and sequence motif families of the maize ear. *PLoS Genet.* **17**, e1009689 (2021).
10. Zhao, H. *et al.* Genome-wide MNase hypersensitivity assay unveils distinct classes of open chromatin associated with H3K27me3 and DNA methylation in *Arabidopsis thaliana*. *Genome Biol.* **21**, 24 (2020).
