# Supplementary material for: Genome-wide modelling of plant transcription factor binding captures regulatory variants associated with phenotypic traits
Source: Nat Commun. 2026 Jun 3;17:4913. doi: 10.1038/s41467-026-73634-8 (PMC13234004; doi:10.1038/s41467-026-73634-8)
Supplement: Supplementary file 3 — Description of Additional Supplementary Files [file 41467_2026_73634_MOESM3_ESM.pdf]

Description of Additional Supplementary Files

Supplementary Data 1. Model performance for individual TF families using non-weighted and weighted loss function training. The performance was evaluated using sensitivity on windows under DAPseq peaks and Mathew's correlation coefficient (MCC), balanced accuracy, f1 micro, f1 weighted and f1 macro scores on genomic windows.

Supplementary Data 2. Performance of unweighted model across 46 TF families including the true Positives (TP), true negatives (TN), false positive (FP) and false (FN)

Supplementary Data 3. Alignment of interaction predictive motifs (IPMs) to known JASPAR motifs. The table shows the top matches between the model-extracted IPMs and experimentally derived transcription factor binding profiles from the JASPAR database. Similarity scores represent the Pearson Correlation Coefficient (PCC), and significance (*p*-values) was determined using a two-sided t-test.

Supplementary Data 4. IPM specific importance scores extracted as SHAPley scores from TF-MoDISco.

Supplementary Data 5. Characterized TFBS PWMs from the JASPAR database have been mapped to the genome of *Arabidopsis thaliana* using FIMO and compared to predicted binding of the TF-family model per 250 bp with a reference PWM to calculate recovery rates.

Supplementary Data 6. Binned numbers and rates of co-occurrences of interaction predictive motifs (IPMs) per transcription factor family.

Supplementary Data 7. Summary statistics of interaction predictive motifs (IPM) predictive importance, window offset independence, weighted co-occurrences, context-importance (IPMciv) and false discovery rates (FDR) by occurrence.

Supplementary Data 8. Evaluation of the occurrence of IPMs and true positive prediction of the TFBS predictive multi-label model.

Supplementary Data 9. MOA peak regions extracted from *Arabidopsis thaliana.*

Supplementary Data 10. Performance of multi-label classifiers evaluated on *Zea mays* (maize) ChIP-Seq and DAP-seq datasets.
