# Supplementary material for: Genome-wide modelling of plant transcription factor binding captures regulatory variants associated with phenotypic traits
Source: Nat Commun. 2026 Jun 3;17:4913. doi: 10.1038/s41467-026-73634-8 (PMC13234004; doi:10.1038/s41467-026-73634-8)
Supplement: Supplementary file 14 — Reporting Summary [file 41467_2026_73634_MOESM14_ESM.pdf]

nature portfolio

Corresponding author(s): Dr. Simon M. Zumkeller, s.zumkeller@fz-juelich.de; NCOMMS-25-23704A

Last updated by author(s): 03-04-2026

Reporting Summary

Nature Portfolio wishes to improve the reproducibility of the work that we publish. This form provides structure for consistency and transparency in reporting. For further information on Nature Portfolio policies, see our Editorial Policies and the Editorial Policy Checklist.

Statistics

For all statistical analyses, confirm that the following items are present in the figure legend, table legend, main text, or Methods section.

n/a	Confirmed	
☐	☒	The exact sample size (*n*) for each experimental group/condition, given as a discrete number and unit of measurement
☐	☒	A statement on whether measurements were taken from distinct samples or whether the same sample was measured repeatedly
☐	☒	The statistical test(s) used AND whether they are one- or two-sided<br>*Only common tests should be described solely by name; describe more complex techniques in the Methods section.*
☐	☒	A description of all covariates tested
☐	☒	A description of any assumptions or corrections, such as tests of normality and adjustment for multiple comparisons
☐	☒	A full description of the statistical parameters including central tendency (e.g. means) or other basic estimates (e.g. regression coefficient) AND variation (e.g. standard deviation) or associated estimates of uncertainty (e.g. confidence intervals)
☐	☒	For null hypothesis testing, the test statistic (e.g. *F*, *t*, *r*) with confidence intervals, effect sizes, degrees of freedom and *P* value noted<br>*Give P values as exact values whenever suitable.*
☒	☐	For Bayesian analysis, information on the choice of priors and Markov chain Monte Carlo settings
☐	☒	For hierarchical and complex designs, identification of the appropriate level for tests and full reporting of outcomes
☐	☒	Estimates of effect sizes (e.g. Cohen's *d*, Pearson's *r*), indicating how they were calculated

Our web collection on statistics for biologists contains articles on many of the points above.

Software and code

Policy information about availability of computer code

Data collection	This section covers all databases, external datasets, and internally generated experimental data used to train, test, and validate the models.Reference Genomes & Annotations:Arabidopsis thaliana (TAIR10): Reference genome, proteome, and annotation sourced from the Ensembl Plants database (release 59).Zea mays (RefGenV4): Reference genome sourced from the Ensembl Plants database (release 50).Transcription Factor (TF) Binding & Motif Databases:PlantCistromeDB: Provided processed DAP-seq peaks for 568 A. thaliana TFs across 46 families.JASPAR2026 Database: Provided annotated motifs from various experimental sources (DAP-seq, ChIP-seq, SELEX, etc.) and biological metadata (TF families, classes, PubMed IDs).NCBI GEO: Sourced Zea mays ChIP-seq and DAP-seq data for cross-species evaluation (Accession numbers: GSE137972, GSE120304, GSE275897).Genetic Variation & Expression Databases:AraGWAS Catalogue: Provided 7,364 single nucleotide polymorphisms (SNPs) associated with traits in A. thaliana.ATTED-II Database: Provided gene co-expression data.Published Experimental Datasets: Deep mutational scan data of the rbcS-E9 enhancer, AB80, and Cab-1 enhancers, as well as promoter strength measurements from ~100 maize core promoters in protoplast assays (referenced from previous studies).Data Generated in This Study:MOA-seq Data: Generated from A. thaliana (Col-0) rosette leaves to map native cistrome occupancy. Deposited in the SRA archive (BioProject PRJNA1225493).Plant STARR-seq Data: Generated using Nicotiana benthamiana leaves to validate the effects of GWAS SNPs on enhancer/promoter strength.
Data analysis	This section covers the software, code repositories, algorithms, and statistical methods used to process the data and build the models.Core Code & Repositories:Custom Code Base: All custom scripts and model code are hosted on GitHub at https://github.com/NAMlab/DeepCistrome.Data Preprocessing & Sequencing Analysis Tools:bedtools: Used to split the genome into 250 bp windows and generate overlap matrices with DAP-seq peaks.samtools (v1.16): Used to determine average fragment lengths for MOA-seq data.unique-kmers.py (khmer): Used to calculate effective genome size.MACS3 (v3.0.0a7): Used for significant peak calling on the MOA-seq data.deepTools (v3.5.0): Specifically the bamCoverage function, used to generate normalized bigwig files (counts per million).Deep Learning Model Architecture & Training:Convolutional Neural Network (CNN): Multi-label classification architecture featuring 4 convolutional blocks (Conv layer, Batch

Normalization, ReLU, MaxPooling) and 2 fully connected blocks (Dense layer, Batch Normalization, ReLU, Dropout), ending with a Sigmoid activation output layer (46 units).Optimization: Adam optimizer (learning rate = 0.002) with early stopping (5-epoch patience).Feature Extraction, Motif Discovery, & Mapping:MLCM (Multi-Label Confusion Matrix): Used to evaluate model performance and identify false positive/negative sources.SHAP: Used to compute nucleotide-resolution importance scores for model predictions.TF-MoDisco: Used in tandem with SHAP to generate Interaction Predictive Motifs (IPMs).motifStack (R package): Used to cluster the generated IPMs based on binding similarities.BLAMM: Used alongside custom R scripts to map extracted IPMs back against chromosome 1 of A. thaliana.FIMO: Used to scan the A. thaliana genome for occurrences of JASPAR motifs (threshold >85% of max theoretical score).Statistical Methods & Logic:Performance Metrics: Sensitivity, micro F1 score, Matthews Correlation Coefficient (derived via 100 bootstrap replicates to handle class imbalance).Mathematical/Statistical Tests: Second-degree polynomial regressions, Z-scores, Fisher's exact test, Benjamini–Hochberg FDR correction (for co-expression data), and paired Wilcoxon tests (for evaluating STARR-seq expression changes).

For manuscripts utilizing custom algorithms or software that are central to the research but not yet described in published literature, software must be made available to editors and reviewers. We strongly encourage code deposition in a community repository (e.g. GitHub). See the Nature Portfolio guidelines for submitting code & software for further information.

Data

Policy information about availability of data

All manuscripts must include a data availability statement. This statement should provide the following information, where applicable:

- Accession codes, unique identifiers, or web links for publicly available datasets
- A description of any restrictions on data availability
- For clinical datasets or third party data, please ensure that the statement adheres to our policy

DAP-seq datasets for Arabidopsis thaliana used for training and validation of our models were downloaded from 19. Zea mays ChIP-seq and DAP-seq data used for cross-species evaluation were downloaded from the NCBI GEO using accession numbers GSE137972, GSE120304 and GSE275897. MOA-seq raw data for Arabidopsis leaves was submitted to the SRA archive under the BioProject PRJNA1225493 (reviewer link:https://dataview.ncbi.nlm.nih.gov/object/PRJNA1225493?reviewer=u3s796pb8suj74mavn5tcvkf4j). Moa peaks are available in narrowPeak format (Suppl. Table 14).

Research involving human participants, their data, or biological material

Policy information about studies with human participants or human data. See also policy information about sex, gender (identity/presentation), and sexual orientation and race, ethnicity and racism.

Reporting on sex and gender	*Use the terms sex (biological attribute) and gender (shaped by social and cultural circumstances) carefully in order to avoid confusing both terms. Indicate if findings apply to only one sex or gender; describe whether sex and gender were considered in study design; whether sex and/or gender was determined based on self-reporting or assigned and methods used. Provide in the source data disaggregated sex and gender data, where this information has been collected, and if consent has been obtained for sharing of individual-level data; provide overall numbers in this Reporting Summary. Please state if this information has not been collected. Report sex- and gender-based analyses where performed, justify reasons for lack of sex- and gender-based analysis.*
Reporting on race, ethnicity, or other socially relevant groupings	*Please specify the socially constructed or socially relevant categorization variable(s) used in your manuscript and explain why they were used. Please note that such variables should not be used as proxies for other socially constructed/relevant variables (for example, race or ethnicity should not be used as a proxy for socioeconomic status). Provide clear definitions of the relevant terms used, how they were provided (by the participants/respondents, the researchers, or third parties), and the method(s) used to classify people into the different categories (e.g. self-report, census or administrative data, social media data, etc.) Please provide details about how you controlled for confounding variables in your analyses.*
Population characteristics	*Describe the covariate-relevant population characteristics of the human research participants (e.g. age, genotypic information, past and current diagnosis and treatment categories). If you filled out the behavioural & social sciences study design questions and have nothing to add here, write "See above."*
Recruitment	*Describe how participants were recruited. Outline any potential self-selection bias or other biases that may be present and how these are likely to impact results.*
Ethics oversight	*Identify the organization(s) that approved the study protocol.*

Note that full information on the approval of the study protocol must also be provided in the manuscript.

Field-specific reporting

Please select the one below that is the best fit for your research. If you are not sure, read the appropriate sections before making your selection.

[x] Life sciences [ ] Behavioural & social sciences [ ] Ecological, evolutionary & environmental sciences

For a reference copy of the document with all sections, see nature.com/documents/nr-reporting-summary-flat.pdf

Life sciences study design

All studies must disclose on these points even when the disclosure is negative.

Sample size	*Describe how sample size was determined, detailing any statistical methods used to predetermine sample size OR if no sample-size calculation was performed, describe how sample sizes were chosen and provide a rationale for why these sample sizes are sufficient.*
Data exclusions	*Describe any data exclusions. If no data were excluded from the analyses, state so OR if data were excluded, describe the exclusions and the rationale behind them, indicating whether exclusion criteria were pre-established.*
Replication	*Describe the measures taken to verify the reproducibility of the experimental findings. If all attempts at replication were successful, confirm this OR if there are any findings that were not replicated or cannot be reproduced, note this and describe why.*
Randomization	*Describe how samples/organisms/participants were allocated into experimental groups. If allocation was not random, describe how covariates were controlled OR if this is not relevant to your study, explain why.*
Blinding	*Describe whether the investigators were blinded to group allocation during data collection and/or analysis. If blinding was not possible, describe why OR explain why blinding was not relevant to your study.*

Reporting for specific materials, systems and methods

We require information from authors about some types of materials, experimental systems and methods used in many studies. Here, indicate whether each material, system or method listed is relevant to your study. If you are not sure if a list item applies to your research, read the appropriate section before selecting a response.

Materials & experimental systems

n/a	Involved in the study	
☒	☐	Antibodies
☒	☐	Eukaryotic cell lines
☒	☐	Palaeontology and archaeology
☒	☐	Animals and other organisms
☒	☐	Clinical data
☒	☐	Dual use research of concern
☐	☒	Plants

Methods

n/a	Involved in the study	
☒	☐	ChIP-seq
☒	☐	Flow cytometry
☒	☐	MRI-based neuroimaging

Plants

Seed stocks	*Report on the source of all seed stocks or other plant material used. If applicable, state the seed stock centre and catalogue number. If plant specimens were collected from the field, describe the collection location, date and sampling procedures.*
Novel plant genotypes	*Describe the methods by which all novel plant genotypes were produced. This includes those generated by transgenic approaches, gene editing, chemical/radiation-based mutagenesis and hybridization. For transgenic lines, describe the transformation method, the number of independent lines analyzed and the generation upon which experiments were performed. For gene-edited lines, describe the editor used, the endogenous sequence targeted for editing, the targeting guide RNA sequence (if applicable) and how the editor was applied.*
Authentication	*Describe any authentication procedures for each seed stock used or novel genotype generated. Describe any experiments used to assess the effect of a mutation and, where applicable, how potential secondary effects (e.g. second site T-DNA insertions, mosiacism, off-target gene editing) were examined.*
